# Ten Simple Rules for Teaching Bioinformatics at the High School Level

**DOI:** 10.1371/journal.pcbi.1002243

**Published:** 2011-10-27

**Authors:** David Form, Fran Lewitter

**Affiliations:** 1 Science Department, Nashoba Regional High School, Bolton, Massachusetts, United States of America; 2 Bioinformatics and Research Computing, Whitehead Institute, Cambridge, Massachusetts, United States of America

Fran Lewitter is Education Editor of *PLoS Computational Biology*.

Given the availability of free, online genomic databases and tools for the analysis of biological data, it is now feasible to teach bioinformatics in the high school classroom [1]. There are a number of reasons why it is appropriate and desirable to introduce bioinformatics at the high school level. Students can engage in inquiry-based activities that involve approaching real-world problems using 21st century skills, while being tailored to high school biology frameworks. Many tools, such as 3-D protein visualization software, allow for differentiated and highly interactive instruction. The foremost reason may be that students can develop a research toolkit that they will be able to use subsequently during college and beyond.

As a high school science teacher for the past 23 years, I (DF) have had the opportunity to incorporate bioinformatics into my courses to enrich the teaching of concepts of molecular biology, human biology, genetics, and evolution, providing increased opportunities for effective differentiated instruction and individual student research. This past experience has inspired the creation of this set of Ten Simple Rules.

It is important to distinguish between curricula designed to teach the fundamentals of bioinformatics and those that utilize bioinformatics as a teaching tool. Examples of both types of successful teaching can be found in Text S1, Text S2, and Text S3.

## Rule 1: Keep It Simple

Set one, or a very few, objectives for each activity. Begin with a few, limited, straightforward goals. For example, an activity may require students to find a limited set of specific information in a GenBank file, such as the coding sequence for a gene, and print it out in FASTA format. You can link these objectives to other, more complicated, concepts in later lessons.

An activity will be more effective if extraneous information is kept to a minimum. The output provided to the students is likely to contain too much information for them to digest during one lesson. Focus on one or a few items.

## Rule 2: Familiarity: Use Activities to Explore Examples That Are Familiar to Students

Familiarity breeds relevance. Much of the information presented to students will be new to them. It will make it easier to understand new concepts or information if they are linked to something that is already familiar to them. High school students are particularly interested in topics that they can relate to their immediate personal or social lives. Choose genes, proteins, or processes that relate to disease, development, or other aspects of human physiology and behavior. Obesity, diabetes, and developmental disorders are some examples that have worked well.

## Rule 3: Link Activities to Preexisting Science Curricula

Bioinformatics exercises are more likely to be used if they are related to the curriculum that is already being taught. In a biology class, a lesson using 3-D protein models is more likely to be utilized if the proteins studied relate to concepts in the curriculum. For example, analysis of hemoglobin structure can be part of units on the circulatory system and genetics (sickle cell disease). The use of 3-D models can be used to help introduce students to structure–function relationships in proteins. Students can utilize 3-D protein models to compare the structures of proteins with very different functions, such as collagen, the estrogen receptor, and alpha amylase.

## Rule 4: Develop Activities That Build on Each Other

More complex tasks and skills can be done successfully if they are broken down into small pieces that are taught separately and then combined in a stepwise fashion. Students can focus on learning one skill or concept at a time.

## Rule 5: Use Activities to Build Skills and to Provide Information through Inquiry-Based Research

Students learn best when the work has meaning and when they are actively pursuing a goal. For example, a student who was asked to find the mRNA sequence for the gene involved in a disease that she was researching was wondering why there were several mRNA sequences for what she thought was a single gene. After an explanation of alternative transcripts and the roles of introns and exons in generating these transcripts, she was excited about her “discovery” and proceeded to explain this to her classmate/friend. She found the concept of RNA editing to be fairly easy because she actively discovered the process as part of her research.

## Rule 6: Provide Opportunities for Individualization

Students will often become more involved if they feel a sense of ownership for their work. Have individual students, or student groups, each research their own gene or protein. For example, each student in a class can be asked to identify the gene and protein associated with a unique genetic disorder. Make sure that the level of difficulty is appropriate for the level and age of the students.

## Rule 7: Address Multiple Learning Styles

Student abilities and learning styles will vary among the class. Make use of the multiple ways that information is presented. For example, the output of BLAST makes use of a colorful graphical interface, a “hit list” in chart format, and sequence alignments. Using all of these will help students to understand a BLAST output.

## Rule 8: Empower Students

Students like solving problems and discovering new information. Allow students to discover the concept or information that you want them to learn. This plays to a real strength of bioinformatics as a teaching tool. Set up activities so that students can follow up and extend their knowledge on their own, using the skills that they have developed.

## Rule 9: Model Processes Using Pen and Paper before Using the Computer

Computers can handle large amounts of data and make complex manipulation of this data in a short period of time—that's why we use them in bioinformatics. However, this can often hide the processes from the students. Have the students run through a simplified mock-up of the data analysis using pencil and paper. For example, have them compare protein sequences and come up with a “score” of relatedness before using a program, such as BLAST (through the NCBI website). Have them find and highlight appropriate data in a printed form of a BLAST readout before they analyze a BLAST readout online by themselves.

## Rule 10: Produce a Product

Have the students use the results of their activity to produce a “product” they can present to the class. If they are researching the structure and function of a protein, have them design a product that uses this protein. For example, in researching leptin they can design an obesity pill.

## Supporting Information

Text S1 **Examples of model curriculum.** Here we provide example curriculum for two types of courses for secondary school students. One is for bioinformatics activities to incorporate in an introductory biology course. The second is for a course “Models for Disease” and is offered to Accelerated/Honors level students after completing a first course in biology.
(DOC)

Text S2 **Example term project for “Models of Disease” class.** For the “Model for Disease” course, students are required to complete a term project that uses bioinformatics tools to study a disease. Here we provide an example presentation given by a student based on their term project.
(PDF)

Text S3 **Tips for developing curriculum.** The materials presented here were also presented as part of a tutorial “Teaching Bioinformatics in High School Biology Courses” held at the International Society for Computational Biology's annual meeting (ISMB) held in Boston, Massachusetts, in July of 2010.
(PDF)
