# Supplementary material for: Ten Simple Rules for Teaching Bioinformatics at the High School Level
Source: PLoS Comput Biol. 2011 Oct 27;7(10):e1002243. doi: 10.1371/journal.pcbi.1002243 (PMC3203051; doi:10.1371/journal.pcbi.1002243)
Supplement: Text S1 — Examples of model curriculum. Here we provide example curriculum for two types of courses for secondary school students. One is for bioinformatics activities to incorporate in an introductory biology course. The second is for a course “Models for Disease” and is offered to Accelerated/Honors level students after completing a first course in biology. (DOC) [file pcbi.1002243.s001.doc]

**Text S1. Examples of Model Curriculum**

Box 1 contains **Examples of Model Curriculum for High School Students.**

The “Models for Disease” curriculum is geared toward Accelerated/Honors level students who have already taken a first course in biology. This course competes with, and provides a stark contrast to, Advanced Placement science courses.  Rather than providing a survey of a large quantity of material, as in AP courses, “Models for Disease” strives to provide students with an opportunity to become comfortable with bioinformatics tools and databases, so that they can conduct their own research. Difficult concepts, such as alternative transcripts and mobile genetic elements are made accessible by becoming a part of student research projects. Depth and application are stressed over breadth.

The content and approach are unique in a high school science setting, at least in our school, so comparison with other approaches on a scientific basis is difficult. Student evaluations have been overwhelmingly positive. I have noticed a markedly increased level of sophistication in the students approach to projects over the course of a semester. This can be seen in the sample presentation that is included with our paper. One student was very proud to have “discovered” alternative transcripts when researching the gene for a genetic disorder. She discovered that the gene could code for two different proteins. This prompted her to review the concept of introns and exons. Her pride was easily observable when she explained her “discovery” to the rest of the class.

The “Models for Disease” curriculum was designed by me (DF) as were the majority of activities used in this class. It could be implemented by other teachers who are familiar with the tools and databases used in this curriculum. However, there is, as yet, not commercially available curriculum with detailed lesson plans and instructions for teachers. Rather, this is presented as a model of how a bioinformatics curriculum can be developed and implemented in the high school classroom.

The activities used in the introductory biology class are used by other teachers in our school and elsewhere. They have been designed to fit in with the Massachusetts State Frameworks for high school biology.

The Accelerated Biology classes are usually four classes of approximately 20 per class. “Models for Disease” has varied from 5 to 17 students .

**Box 1.**

**Examples of Model Curriculum for High School Students.**

A. Using bioinformatics as a teaching tool within an introductory high school biology class.

Activities are included in the standard high school biology curricula that utilize bioinformatics tools to reinforce concepts that are currently part of the state and national frameworks. This approach can help teachers to develop inquiry-based lessons and to differentiate the ways in which material is presented to their students. “Recreating the Tree of Life Using Bioinformatics” is one such lesson. This activity has students taking rRNA sequences from various organisms and producing a phylogenetic tree based upon these sequences. The phylogenetic tree involves the use of CLUSTAL W and DRAWGRAM which are available through the Biology Workbench (http://workbench.sdsc.edu/) which is available online through the San Diego Supercomputer Center. Teachers and students have the option of analyzing their data suing a distance matrix in addition to the phylogenetic tree. The phylogenetic tree that they produce is used to discuss the division of living organisms into three domains. Both phylogenetic trees and the three-domain classification system are a part of the introductory biology curriculum. These lessons were previously created as part of the Life Sciences-Howard Hughes Medical Institute Outreach Program at Harvard University and are available online (see Microbiology -> Lesson Plans -> Recreating the Tree of Life Using Bioinformatics – http://outreach.mcb.harvard.edu/materials.htm). Tips for developing and implementing these types of activities are provided in Text S3.

B. An Elective Course focusing on Bioinformatics

One of us (DF) has developed and taught an elective "Models for Disease", a course that focuses on bioinformatics. In this course, inquiries into specific diseases are used to teach the relationships between genes, proteins and disease. Students learn to use various bioinformatics databases and tools to research genetic and infectious diseases and are introduced to concepts in genomics and molecular biology as well as various conceptual models for understanding the genetic basis of disease. Then students learn to apply these concepts and tools to their own research by focusing on specific genetic disorders and infectious diseases. The course concludes with an independent student research project.

In the introductory unit, students learn to access the genomes of various viruses using the National Center for Biotechnology (NCBI) website (http://www.ncbi.nlm.nih.gov/). Students build a model of HIV-1 and relate the gene and protein sequences that they have found to structures and functions in HIV-1. This introduces them to some of the basic vocabulary of genomics, and the genome –associated information that can be located in a NCBI Genome flat file. The class presentation includes a link to the NCBI Genome flat file for HIV-1. Strategies for combating HIV are discussed in light of the specific genes and proteins that are targeted. Students are then asked to use NCBI Genome to research if any of these strategies might apply to other viruses, based on the proteins encoded in their genomes.

The following unit of Models for Disease focuses on genetic disorders. This encourages the students to develop an understanding of recessive, dominant and sex-linked disorders. Students learn to access information from NCBI (<http://www.ncbi.nlm.nih.gov/>), GeneCards (<http://www.genecards.org/>) and The Genetics Home Reference (<http://ghr.nlm.nih.gov/>). They learn to use BLAST in order to identify suitable laboratory models for the study of individual diseases. The use of online bioinformatics tools and the genomics concepts associated with the use of these tools is presented in a graded manner. In this way, students build up a bioinformatics skillset and a genomics knowledge base that they can use to investigate topics that are of interest to them.

By the end of the first unit, the focus shifts to protein structure and function. Students learn to align and compare protein sequences, using a variety of online bioinformatics tools. They research a sweet protein, monellin, and work in groups to design a new sweetener based on analyzing research done on this protein, using tools such as BLAST and PubMed.

The second half of "Models for Disease” focuses on infectious disease. A project involving 3-D (3 dimensional) visualization of bacterial toxins is presented as an example from this part of the course. Expanding the focus on protein structure and function, students study cholera toxin using J-mol, a 3-D modeling software package. A link to this model is provided in the slide presentation (see Supplementary information). Students use BLAST and 3-D protein models to compare the primary, secondary, tertiary and quaternary structure of the cholera toxin with that of a toxin derived from atwo different pathogenic strains of E. coli. The class studies various mechanisms involved in the horizontal transfer of genes in bacteria and relate this to the evolution of new diseases. They conclude their project by identifying an island of pathogenicity in the Vibrio cholera genome.

Students learn to use CLUSTALW to make multiple alignments of protein sequences and they utilize an online tool, DrawGram, to construct phylogenetic trees, based on these alignments. These tools are utilized by the students to determine the origin of HIV-1 and HIV-2.

“Models for Disease” concludes with an independent student research project. The students prepare a slide presentation and a written research paper. Students use the tools presented in this class to research a genomics topic of their choice. Some topics have included genes involved in alcoholism, the genetic basis for lactose intolerance and the evolutionary origin of the plague.
