# Supplementary material for: Ten Simple Rules for Teaching Bioinformatics at the High School Level
Source: PLoS Comput Biol. 2011 Oct 27;7(10):e1002243. doi: 10.1371/journal.pcbi.1002243 (PMC3203051; doi:10.1371/journal.pcbi.1002243)

Wilson Disease

By xxxxx

What is Wilson's disease?

- An **inherited** disorder
- Excessive amounts of **copper** accumulate in the body
  - **liver**
  - **brain**
  - **eyes**

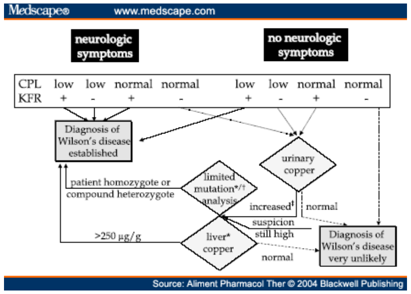

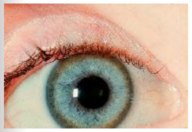

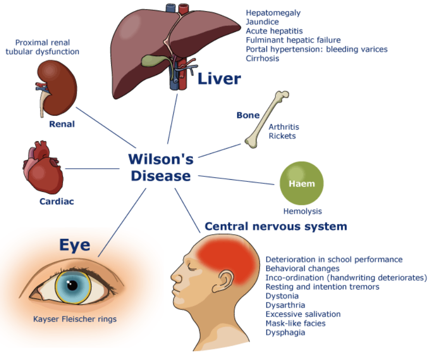

Symptoms

- **Yellowing** of the skin or the whites of the eye (jaundice)
- **Fatigue**
- **Loss of appetite**
- **Abdominal swelling**
- **Psychiatric** or **nervous** system problems in **young adults**
- **Clumsiness, trembling, difficulty walking, speech problems, deteriorating school work, depression, anxiety,** and **mood swings**
- Copper deposits form a green-to-brownish ring, called the **Kayser-Fleischer ring**, around cornea (in eye)
- **Abnormalities in eye movements**

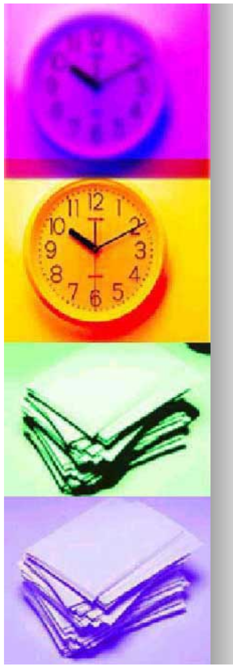

Who is affected

- **1 in 30,000** individuals
- Signs and symptoms first appear between the ages of **6** and **40**, but most often begin during the **teenage years**

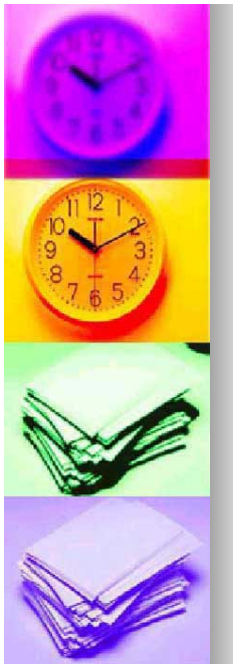

How is it inherited?

- **Autosomal** recessive pattern
  - **both** copies of the gene in each cell have mutations.
  - Parents of an individual with an autosomal recessive condition **each** carry one copy of the mutated gene
    - They typically do **not** show signs and symptoms of the condition

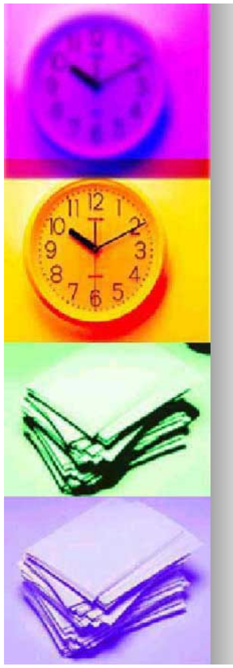

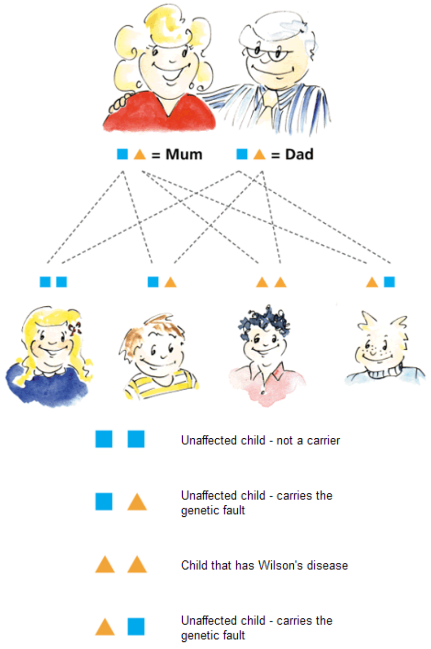

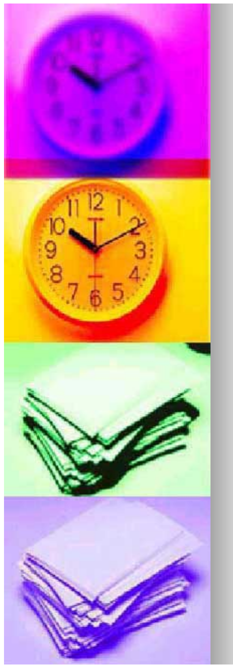

Mutations

- Mutations in the ATP7B gene cause this disease.
- Normal variations in the PRNP gene modify the course of Wilson disease.
- The ATP7B gene
  - provides instructions for making a protein
    - Plays a role in the transport of copper from the liver to other parts of the body.
    - Particularly important for the elimination of excess copper from the body.
  - Mutations in the ATP7B gene prevent the transport protein from functioning properly
    - Due to the shortage of functional protein, excess copper is not removed from the body.
    - Copper accumulates to toxic levels that can damage tissues and organs, particularly the liver and brain.
    - This explains why the symptoms occur
- A normal variation in the PRNP gene may delay the age of onset of Wilson disease and affect the type of symptoms that develop.

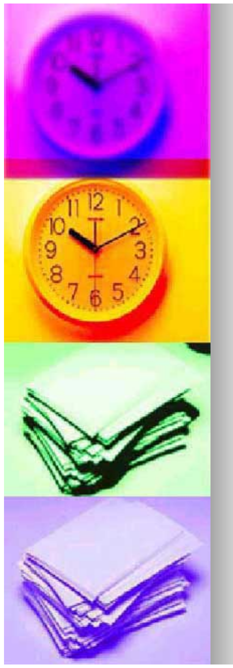

PRNP: More Details

- The PRNP gene provides instructions for making prion protein, which is active in the brain and other tissues.
- Transferring copper
  - Studies have focused on the effects of a PRNP gene variation that affects the prion protein at position 129.
    - At this position, the protein building block (amino acid) methionine or valine is used.
- Onset of symptoms of Wilson disease is delayed by several years if person has methionine (instead of valine) at position 129 in the prion protein.
  - Also may increase symptoms that affect the nervous system (tremors).
- Larger studies needed to establish effects of this PRNP variation

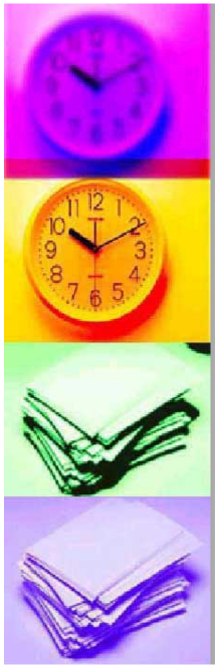

Charts

- **Genomic regions, transcripts, and products**

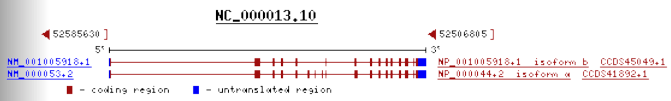

- Genomic context

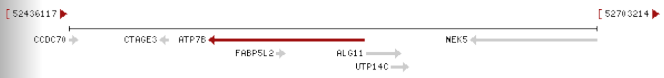

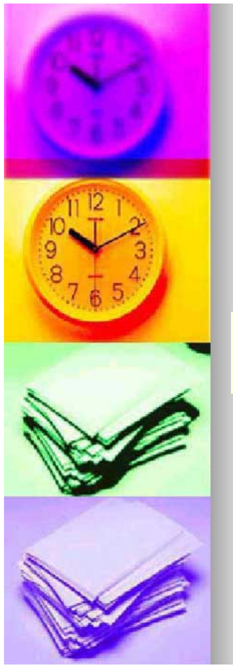

Chromosome

- **Chromosome:** 13
- **Location:** 13q14.3

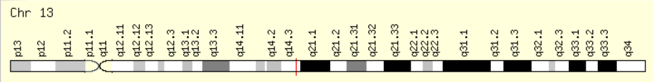

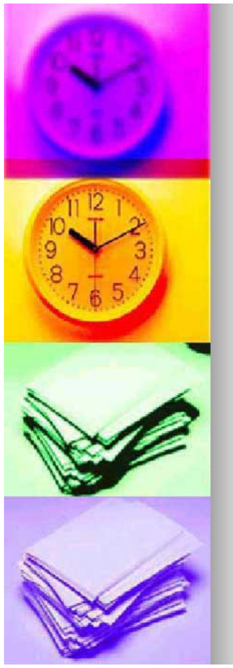

NCBI links, etc

- Link for the gene, ATP7B:
- http://www.ncbi.nlm.nih.gov/gene/540?ordinalpos=2&itool=EntrezSystem2.PEntrez.Gene.Gene_ResultsPanel.Gene_RVDocSum
- Link to FASTA for one of the two sequences

http://www.ncbi.nlm.nih.gov/nuccore/NM_001005918.1?report=fasta

That's for NM_001005918.1. The other is NM_000053.2.

Protein sequence:

http://www.ncbi.nlm.nih.gov/protein/119722350?report=fasta&log$=seqview

Involved in the **export of copper out of the cells**

Animal to study

- This disease can be studied in **rats**
- The e-value is **2e-13**
- When you compare the protein sequences using **BLAST**, you find that the human protein sequence is **homologous** with the sequence in rats
- Although this is not very low of a value, the sequences are **95% identical**, which makes me confident that this would be an appropriate test animal
- The reason the e-value is so low is because the **protein sequence is short**

Diagrams

- Provided by http://www.hcforum.fr/eurowilson/?page=0&langue=it

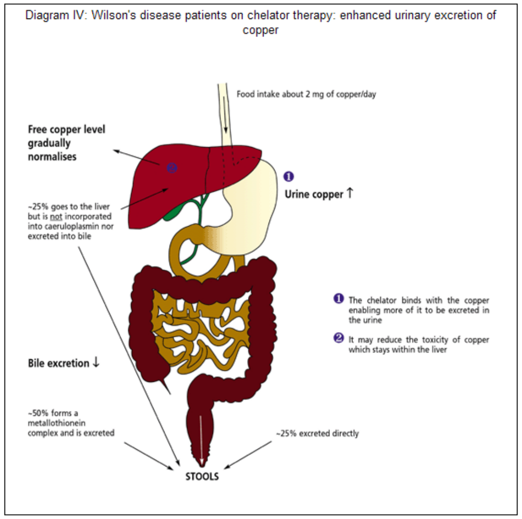

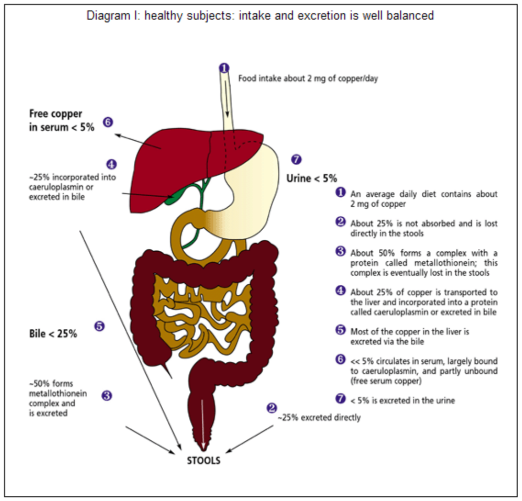

Diagram II: Wilson's disease patients before treatment: reduced excretion and retention of copper

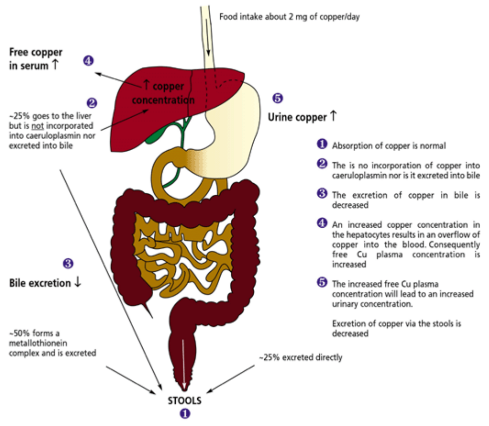

Diagram III: Wilson's disease patients on zinc therapy: enhanced faecal excretion of copper

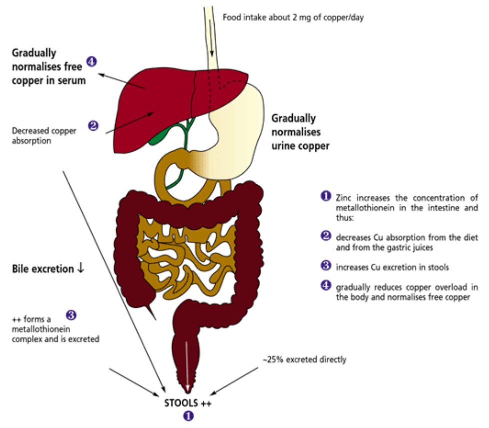

More information:

- Treatment
  - American Association for the Study of Liver Disease: Diagnosis and Treatment of Wilson Disease
  - Gene Reviews
  - MedlinePlus Encyclopedia: Wilson's disease
- MedlinePlus - Health information (2 links)
- Additional NIH Resources - National Institutes of Health (3 links)
- Educational resources - Information pages (8 links)
- Patient support - For patients and families (5 links)
- Gene Reviews - Clinical summary
- Gene Tests - DNA tests ordered by healthcare professionals
- ClinicalTrials.gov - Linking patients to medical research
- PubMed - Recent literature
- Online Books - Medical and science texts
- Scriver's Online Metabolic and Molecular Bases of Inherited Disease (OMMBID): Disorders of Copper Transport
- OMIM - Genetic disorder catalog
- More questions?
  - See How can I find a genetics professional in my area? in the Handbook.
  - Ask the Genetic and Rare Diseases Information Center .
  - Submit your question to Ask the Geneticist .
  - What does it mean if a disorder seems to run in my family?
  - What are the different ways in which a genetic condition can be inherited?
  - If a genetic disorder runs in my family, what are the chances that my children will have the condition?
  - Why are some genetic conditions more common in particular ethnic groups?
  - Genetics and health
  - Resources for Patients and Families
  - Resources for Health Professionals

Links provided by Genetics Home Reference

Bibliography

- http://www.ncbi.nlm.nih.gov/
- http://www.hcforum.fr/eurowilson/?page=0&langue=it
- http://ghr.nlm.nih.gov/
- http://www.genecards.org/

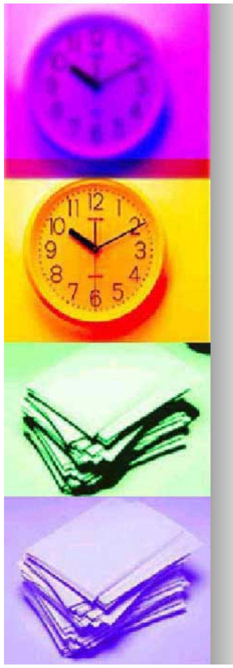

The End!

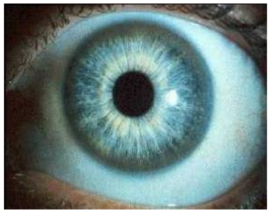

Supplement: Text S2 — Example term project for “Models of Disease” class. For the “Model for Disease” course, students are required to complete a term project that uses bioinformatics tools to study a disease. Here we provide an example presentation given by a student based on their term project. (PDF) [file pcbi.1002243.s002.pdf]
