# Supplementary material for: Ten Simple Rules for Teaching Bioinformatics at the High School Level
Source: PLoS Comput Biol. 2011 Oct 27;7(10):e1002243. doi: 10.1371/journal.pcbi.1002243 (PMC3203051; doi:10.1371/journal.pcbi.1002243)

Teaching Bioinformatics in the High School Classroom

David Form
Nashoba Regional High School
dform@nrsd.net

Why teach bioinformatics in high school?

- Relevant, real life examples
- It's visual
- Allows for inquiry based approach
- Allows for personalization
- Allows for reinforcement of basic concepts
- It's cheap, with no cleanup required!

Students Develop a Research Tool Kit That Will be Useful in College

- Access information from online genomics databases
- Use BLAST to
  - Compare Genes and Proteins
  - Find model organisms
- Use PubMed to find relevant papers

Models for Disease

A high school level genomics/ bioinformatics course

Goals

- Develop an understanding of the relationship between genes, proteins and disease
- Learn to ask the types of questions that are asked by scientists
- Conduct independent genomics-based research
- Develop a toolkit of online bioinformatics tools that they can use in college or in a laboratory setting

Models for Disease Presents a series of case studies

- Specific diseases are used as models for investigating the relationships between genes, proteins and disease
- Students apply newly acquired skills towards independent research.

3 Strands Taught in Parallel

- How to use various online bioinformatics databases and tools
- Genomics and molecular genetics
- Genetic basis of disease

The Curriculum

Introductory Unit

Modeling the Genome of HIV

The Genome of HIV

- Students construct
  1. A 3-D model of HIV
  2. A poster containing
     - The complete genome of HIV
     - Function and nucleotide sequence of each of the genes
     - Function and amino acid sequence of major proteins
- Students relate structures in 3-D model to specific genes and proteins in their genomic poster
- Students produce an online genomic scavenger hunt for a different virus

Goals for Unit

- Associate events in life cycle of HIV with specific
  – Structures
  – Genes
  – Proteins
- Learn to use NCBI Genome to find genomic information

- NCBI Genome for HIV 1
- (http://www.ncbi.nlm.nih.gov/sites/entrez?Db=genome&Cmd=ShowDetailView&TermToSearch=12171)

Part I

Modeling Genetic Disorders

Genetic Disorders

- **Genetics**
  - Structure of genomes
    - Introns/exons
    - mRNA structure
    - Promoters
    - Coding and non-coding sequences
  - Recessive disorders
  - Dominant disorders
  - Sex-linked disorders
  - Complex disorders
    - Aging and obesity
- **Bioinformatics**
  - Use of genomic databases
    - NCBI, GeneCards, Genetics Home Reference
    - BLAST
      - Finding suitable laboratory models for disease

First Major Culminating Assignment

- Choose a genetic disorder
- Symptoms
- Gene(s) involved
  - Nucleotide sequence
  - Chromosomal address
  - Number of alternative transcripts
  - Gene expression profile
- Protein encoded by the gene
  - Function and relationship to symptoms
  - Amino acid sequence
- Use BLAST to find a suitable laboratory model for this disorder

Research limited to three resources

- Genetics Home Reference
- Gene Cards
- NCBI

Example of Student Work

Wilson's Disease

(see accompanying PDF for student's work)

Bridge

The Monellin Project

Designing a Novel Sweetener: The Monellin Project

- Students work in groups to design a bioengineered protein for use as a natural non-nutritive sweetener
  - Demonstrate uniqueness of their protein
  - Compare natural monellin with genetically engineered monellin
  - Alter the active site to improve sweetness

Part II

Modeling Infectious Disease

Infectious Disease

- **Units**
  - **Cholera and the Germ Theory of Disease**
    - The cholera toxin
    - Transmission of disease through water
  - **Outbreaks of pathogenic E. coli**
    - Lateral transfer of toxins and the evolution of disease
    - Mechanisms of gene transfer between bacteria
  - **HIV**
    - Evolutionary origin of HIV
    - The case of the Florida dentist
    - CCR5 gene and resistance to AIDS
- **Bioinformatics**
  - Comparisons of Protein Structure
    - Aligning primary structures of proteins
    - 3-D models of proteins
    - Domains
  - Multiple Alignments
  - Constructing phylogenetic trees

Comparison of cholera toxin with E. coli enterotoxin

Using 3-D protein modeling software

And BLAST

- FirstGlance in Jmol
- (http://molvis.sdsc.edu/fgij/)

Part III

Independent Research Project

Final Project: Independent Student Research

- PowerPoint and Research Paper
  - Must use tools covered by this class
  - Some topics
    - Genes involved in
      - Speech
      - Intelligence
      - Alcoholism
    - Genetic basis for lactose intolerance
    - Evolutionary origin of the plague

Incorporating Bioinformatics into the Introductory Biology Curriculum

Goals

- Introduce students to genomics databases and tools
- Reinforce topics/concepts in curriculum
- Personalize the curriculum
- Application to real life topics
- Empower students by using online tools

Activities

- Evolution of whales
- The vitamin C pseudogene as molecular evidence for evolution
- rRNA and the three domains of life
- What is a Tasmanian wolf?
- Homeotic genes and the evolution of eyes

Whales on Trees

Using DNA evidence to help unravel the ancestry of whales

Mesonychids: Wolf-like or Wolf-Related?

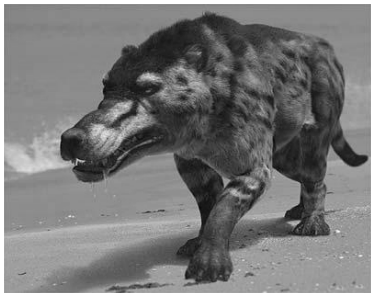

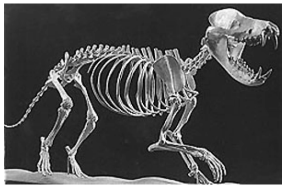

Students use DNA sequences to construct phylogenetic trees in order to determine the closest living relatives of the whales

Relationship of whales to various mammals

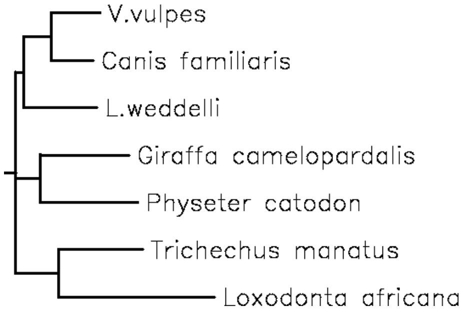

Relationship of whales to various ungulates

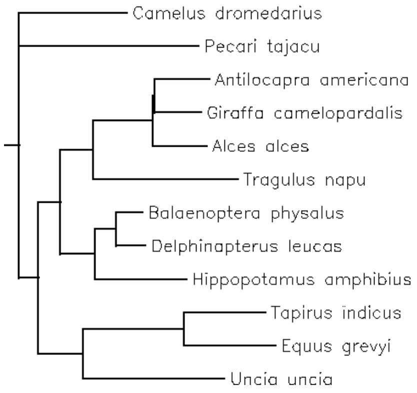

Supplement: Text S3 — Tips for developing curriculum. The materials presented here were also presented as part of a tutorial “Teaching Bioinformatics in High School Biology Courses” held at the International Society for Computational Biology's annual meeting (ISMB) held in Boston, Massachusetts, in July of 2010. (PDF) [file pcbi.1002243.s003.pdf]
